# Knowledge integration in One Health policy formulation, implementation and evaluation

**DOI:** 10.2471/BLT.17.202705

**Published:** 2018-02-05

**Authors:** Martin Hitziger, Roberto Esposito, Massimo Canali, Maurizio Aragrande, Barbara Häsler, Simon R Rüegg

**Affiliations:** a Epidemiology Section, Vetsuisse Faculty, University of Zurich, Winterthurerstrasse 270, 8057 Zürich, Switzerland.; b External Relation Office, National Institute of Health, Rome, Italy.; c Department of Agricultural and Food Sciences, University of Bologna, Bologna, Italy.; d Department of Pathobiology and Population Sciences, Royal Veterinary College, London, United Kingdom.

## Abstract

The One Health concept covers the interrelationship between human, animal and environmental health and requires multistakeholder collaboration across many cultural, disciplinary, institutional and sectoral boundaries. Yet, the implementation of the One Health approach appears hampered by shortcomings in the global framework for health governance. Knowledge integration approaches, at all stages of policy development, could help to address these shortcomings. The identification of key objectives, the resolving of trade-offs and the creation of a common vision and a common direction can be supported by multicriteria analyses. Evidence-based decision-making and transformation of observations into narratives detailing how situations emerge and might unfold in the future can be achieved by systems thinking. Finally, transdisciplinary approaches can be used both to improve the effectiveness of existing systems and to develop novel networks for collective action. To strengthen One Health governance, we propose that knowledge integration becomes a key feature of all stages in the development of related policies. We suggest several ways in which such integration could be promoted.

## Résumé

Le concept «Un monde, une santé» a trait aux corrélations entre la santé humaine, la santé animale et l'environnement, et requiert la collaboration de différentes parties prenantes sur de nombreux plans culturels, disciplinaires, institutionnels et sectoriels. Or, la mise en œuvre de ce principe est rendue difficile par des défauts du cadre mondial de gouvernance en matière de santé. Les approches qui visent à regrouper les connaissances, à toutes les étapes de l'élaboration des politiques, pourraient permettre de résoudre ces défauts. Des analyses multicritères pourraient contribuer à définir des objectifs clés, à résoudre les compromis et à créer une vision et une direction communes. Une pensée systémique pourrait déboucher sur une prise de décisions d’après des éléments probants et transformer les observations en descriptions détaillant la manière dont des situations surviennent et pourraient évoluer dans l'avenir. Enfin, des approches transdisciplinaires pourraient permettre d'améliorer l'efficacité des systèmes existants tout en développant de nouveaux réseaux d'action collective. Afin de renforcer la gouvernance du principe «Un monde, une santé», nous proposons que le regroupement des connaissances devienne un élément clé de toutes les étapes de l'élaboration des politiques relatives à ce principe et suggérons plusieurs manières de favoriser ce regroupement.

## Resumen

El concepto de One Health cubre la interrelación entre la salud humana, animal y ambiental, y exige la colaboración de varias partes interesadas atravesando diversos límites culturales, disciplinarios, institucionales y sectoriales. Sin embargo, la implementación del enfoque de One Health parece verse obstaculizado por deficiencias en el marco global de la gobernanza sanitaria. Los enfoques de integración de conocimientos, en todas las etapas del desarrollo de la política, podrían contribuir a abordar estas deficiencias. Los análisis basados en numerosos criterios permiten respaldar la identificación de objetivos claves, la resolución de dilemas y la creación de una visión común y una dirección común. El pensamiento sistémico puede lograr la toma de decisiones basadas en pruebas y la transformación de las observaciones en textos donde se describa detalladamente cómo surgen las situaciones y cómo estas podrían desarrollarse en el futuro. Por último, pueden emplearse enfoques transdisciplinarios para mejorar la efectividad de los sistemas existentes y desarrollar redes innovadoras para la acción colectiva. A fin de fortalecer la gobernanza de One Health, proponemos que la integración de conocimientos se convierta en un aspecto clave de todas las etapas del desarrollo de las políticas relacionadas con One-Health. Sugerimos diferentes maneras de promover dicha integración.

## ملخص

يغطي مفهوم "توحيد الأداء في مجال الصحة" العلاقة المتبادلة بين البشر والحيوانات والصحة البيئية، ويتطلب ذلك تعاون الجهات المعنية العابر لحدود الثقافات والتخصصات والمؤسسات والقطاعات. ومع ذلك يبدو أن تنفيذ نهج "توحيد الأداء في مجال الصحة" تعرقله أوجه للقصور في الإطار العالمي للإدارة الصحية. ويمكن للأساليب المنهجية دمج المعلومات المساعدة في معالجة جميع أوجه القصور في جميع مراحل تطوير السياسات. ويمكن أن يتم دعم الجهود الرامية إلى تحديد الأهداف الأساسية، وإيجاد حلول المفضالات، وخلق رؤية مشتركة، وتوجه مشترك من خلال إجراء تحليل متعدد المعايير. ويمكن تحقيق عملية اتخاذ القرارات القائمة على أدلة، وتحويل الملاحظات إلى معلومات سردية تشرح بالتفاصيل كيفية ظهور الحالات واكتشافها في المستقبل من خلال التفكير المنهجي. وأخيرًا، يمكن اتباع الأساليب المنهجية متعددة التخصصات لتطوير فاعلية الأنظمة القائمة، وكذلك لتطوير شبكات جديدة من أجل التحرك الجماعي. وحرصًا على تعزيز الإدارة الصحية الواحدة، فإننا نقترح أن يصبح دمج المعرفة سمة أساسية في جميع مراحل تطوير السياسات ذات الصلة. قمنا باقتراح عدة طرق يمكن من خلالها تشجيع ذلك الدمج.

## 摘要

“唯一健康”概念涵盖了人类健康、动物健康和环境健康之间的相互关系,需要多个利益相关方在诸多文化、学科、机构和部门之间的合作。然而,“唯一健康”的实施方法似乎受到全球卫生治理框架中的缺陷的阻碍。知识整合方法在政策制订的各个阶段都可以帮助解决这些缺点。关键目标的确定、权衡的解决以及建立一个共同愿景和展开叙述可以通过多重判据分析来实现。通过系统性思考,可实现基于证据的决策并将观察结果转化为对未来形势会如何出现及展开的详细叙述。最后,跨学科方法既可以用来提高现有系统的有效性,也可以用来开发全新的集体行动网络。为了加强“唯一健康”的治理,我们建议将知识整合作为“唯一健康”相关政策发展过程中的一个关键特征。我们提出几种可以促进这种整合的方法。

## Резюме

Концепция «Один мир — одно здоровье» охватывает взаимосвязь между здоровьем человека, животных и состоянием окружающей среды и требует многостороннего сотрудничества во многих культурных, дисциплинарных, институциональных и секторальных областях. Тем не менее внедрению подхода «Один мир — одно здоровье», вероятно, препятствуют недостатки в глобальной структуре управления здравоохранением. Подходы с применением интеграции знаний на всех этапах разработки политики могут помочь устранить эти недостатки. Выявление ключевых целей, урегулирование компромиссов и выработка общего видения и общего направления деятельности могут быть подкреплены многокритериальными анализами. Принятие решений на основе фактических данных и преобразование наблюдений в нарративы с подробным описанием того, как возникают и могут развиваться ситуации в будущем, могут быть достигнуты путем системного мышления. Наконец, трансдисциплинарные подходы могут использоваться как для повышения эффективности существующих систем, так и для разработки новых сетей для коллективных действий. Для лучшего управления политикой «Один мир — одно здоровье» авторы предлагают сделать интеграцию знаний ключевой особенностью всех этапов разработки этой политики. Предлагается несколько возможных способов содействия такой интеграции.

## Introduction

The concept of One Health initially arose from integrated research on zoonoses,^1^^,^^2^ but now covers all of the interconnections between human, animal and environmental health. The concept is a collaborative, interdisciplinary and intersectoral multi-institutional approach, linking many different forms of knowledge and expertise.^3^^–^^5^ One Health is represented by a complex biological and social system that involves multiple actors and processes and their interactions over time, at local, national and global levels.^5^ To date, relatively little attention has been given to the epistemological, institutional, political and social factors associated with the implementation of a One Health approach.^2^^,^^6^ This is illustrated by the almost complete lack of literature on One Health governance.

There is an existing framework for global health governance: a combination of the formal and informal institutions, rules and processes that influence global decisions on health policy.^7^^,^^8^ Ideally, such a framework should transcend national boundaries, embrace multisectoral and interdisciplinary approaches and engage with the whole wide range of relevant actors.^9^ In reality, however, the current framework is affected by fragmentation of health interests, programmes and sectors, a general lack of societal participation and by professional focus on very limited areas of expertise, so-called professional silos.^10^^,^^11^ The dysfunctionality of the current framework, in terms of the core elements of the One Health concept, emphasizes the need for a dedicated framework for One Health governance.^12^

It has been suggested that some of the current framework’s shortcomings could be overcome by the development of coordinated supranational bodies, the promotion of specialized training and career opportunities and the creation of dedicated funding mechanisms.^9^^,^^12^^–^^14^ We suggest that the framework may also be strengthened by improving the integration of its management^4^^,^^7^^,^^15^ and by integrating knowledge at all stages of any related policy development.^16^^–^^18^ In 2012, knowledge integration was listed as one of the United States National Cancer Institute’s key recommendations for improving 21st century epidemiology.^19^

Since 2014, about 230 experts and representatives of governments and nongovernmental organizations, from the fields of environmental, public and veterinary health and associated sciences, have come together in the Network for Evaluation of One Health.^20^ This network’s main aim is to develop standards for assessing integration in One Health. Since 2016, this work has been enhanced by a core group of experts on complex systems, governance and knowledge integration. This paper summarizes the results of this group’s investigation of knowledge integration in governance, as a mechanism for multi-institutional learning to improve the governance and coordination of One Health implementation in the absence of hierarchical chains of command.

## Coordination and governance

In policy cycles, multiple rounds of agenda setting, policy formulation, decision-making, implementation and evaluation lead to the creation, implementation and revision of policies.^21^ We believe that, in terms of the interdisciplinary, intersectoral and multi-institutional One Health approach, knowledge integration at every stage of policy development, in every policy cycle, could strengthen the coordination and governance of One Health implementation. Although some integration of knowledge from different disciplines, institutions and sectors can, and does, take place intuitively, in many circumstances, we believe that it needs to become a regular, routine and institutionalized process at project, programme and policy levels.^8^^,^^18^^,^^22^ In the development of health policies, knowledge assessment is often confined to the last, that is evaluation, stage of each policy cycle.^23^ We believe that, to optimize the coordination and governance of the One Health approach, knowledge integration should be central at every stage of policy development.

In its broadest sense, knowledge integration has been defined as the building of shared and meaningful syntheses between distinct mental models, based on a recognition and explanation of the relevant differences between the models.^24^^,^^25^ Rather than seeking consensus, knowledge integration can be used to build a common framework that allows an understanding of the links between the knowledge of multiple individuals. Such integration has been likened to the weaving of multiple perspectives into a central vision or a search for coherence and correspondence.^26^^,^^27^ The fostering of effective knowledge integration in a policy cycle is a multidimensional challenge because it requires the integration of cognitive concepts, organizational and social interests and perspectives as well as communicative and cultural factors. The relevant literature distinguishes target knowledge from systems and transformation knowledge. Target, or normative, knowledge relates to objectives and interests, while systems, or descriptive, knowledge relates to perspectives on factual processes. Transformation, or prescriptive, knowledge relates to the transformation of the current version of a system towards a more desired version.^28^ The integration of these three forms of knowledge throughout a policy cycle can be facilitated by three different approaches: multicriteria analyses for target knowledge, systems thinking for systems knowledge and transdisciplinary approaches for transformation knowledge.

### Multicriteria analyses

The key to integrating target knowledge is to understand the often-conflicting interests, preferences and values of the multiple actors, as a first step to mediation, negotiation and, ultimately, collective action.^29^ Multicriteria analyses can assist such integration because they elicit and structure value systems in a way that accommodates a multiplicity of information sources and types.^30^ Such analyses can incorporate any objective that has relevance to the point of view under consideration, rely on non-monetary units and apply valuation methods that are independent of pricing mechanisms. This makes these analyses particularly suited for priority setting in implementation of the One Health approach, which typically involves equity, intergenerational justice and non-marketed goods.^31^^,^^32^ When combined with systems analysis for strategic, long-term assessments, multicriteria analyses offer a flexible yet systematic method of valuation that can bridge the gap between governance and action.^33^^,^^34^

### Systems thinking

Systems knowledge refers to an understanding of the complex interactions, between the many actors and processes in the fields of human, animal and environmental health that emerge and feed back over long time scales. To integrate such knowledge, the management discipline known as systems thinking can be used. Systems thinking can assist human thought by permitting the analytical inference of dynamic consequences, from complex nets of long causal chains that often have feedback loops and unintended effects. System thinking also allows information from multiple sources, e.g. quantitative data, expert knowledge and stakeholders’ experiential insight, to be combined systematically.^35^^,^^36^ These different sources of information are complementary because of missing data, methodological differences and interest-based selective perception, even among members of the same scientific team.^37^^,^^38^ By using all of the available relevant information to understand the possible outcomes of policy interventions and by linking diverse bodies of relatively abstract information with the narratives that guide everyday experience, systems thinking can reduce uncertainty in complex governance problems.^15^^,^^39^^,^^40^

### Transdisciplinarity

Transdisciplinary approaches, which are sometimes called boundary management, are designed to build a bridge, at the science–policy interface and between potentially diverse knowledge systems, by facilitating communication, mediation and translation across cultural, disciplinary, institutional and/or sectoral divides. Although multiple analytic methods may be employed,^29^^,^^37^ the distinctive characteristics of such approaches are mainly sociocultural and aim to foster collective action towards societal transformations.^38^^,^^41^^,^^42^ They include the selection of actors that legitimately represent the interest groups of relevance to the research problem. Co-leadership helps to ensure the equitable representation of interests and perspectives and to mitigate power differentials. The joint negotiation and definition of research objectives and hypotheses is a crucial step in linking target knowledge, building mutual understanding and enabling successful collaborations. Linking narratives and experiential perceptions with conceptual or explanatory, systems knowledge is a central challenge. This challenge can be overcome by careful consideration and the development of a deep understanding in experiential encounters, by repeatedly exposing the different bodies of knowledge to each other and by working towards joint outputs. The sustained commitment of the varied stakeholders needs to be supported by strong leadership, trust building and conflict management.^28^^,^^29^^,^^37^ Transdisciplinary approaches may make three crucial contributions to societal transformations. First, they create social contexts for successful knowledge integration, even where such contexts do not occur naturally. Second, as a result of their collaborative and interactive nature, they tend to produce knowledge that is generally perceived as credible, legitimate and salient. Finally, by fostering collaboration among societal and scientific partners, they can build trust and networks that are independent of any hierarchical chains of command.

## Case studies

We believe that the effective implementation of the One Health strategy, as an interdisciplinary and intersectoral approach that links different forms of knowledge and expertise across multiple institutions, depends on knowledge integration. Six case studies support this view: three general One Health initiatives and three integrated health initiatives that included multicriteria analyses, systems thinking or a transdisciplinary approach (Table 1).

**Table 1:** Comparison of integration of three types of knowledge in six initiatives

Initiative, country, study period	General details	Integration		
		Systems knowledge	Target knowledge	Transformation knowledge
**One Health initiatives**				
West Nile Virus surveillance, Italy, from 2013^43^	Inter-institutional working groups of local and regional authorities in human, animal and environmental health, covering Emilia-Romagna, Lombardy and Piedmont. Implementation of integrated surveillance of birds, horses, humans and mosquitos, including sampling protocols, technical procedures, data-sharing agreements and public information campaigns.	Comprehensive conceptual framework, multispecies sampling protocols, data sharing and linking of information in interdisciplinary groups allowed for integration of systems knowledge. Dissemination to the general public promoted via seminars and educational activities.	Shared leadership fostered integration of target knowledge. Objectives and targets, for overall initiative and individual expert teams, were well defined. Lack of funding for specific targets demonstrated the incomplete alignment of objectives between central and local levels. Institutional set-up lacked flexibility for adaptation.	Strong institutional backing and complex and competent actor network facilitated legitimacy, implementation and resilience. Joint field activities created a team spirit and fostered communication. Annual plenary meetings improved effectiveness. However, public involvement and accessibility of transformation knowledge were considered limited.
Opisthorchiasis control in Lawa province, Thailand, from 2005^44^	Longstanding research track at local university complemented with community-based integrated surveillance, parasite sampling in fish, human screening, medical treatment and education campaigns targeted at public and schools. Linked to international helminth control programme.	Research on opisthorchiasis endemicity and human prevalence. Collaboration with community members for data collection and dissemination fostered integration of local systems knowledge. The need for a more integrated surveillance approach, to understand transmission dynamics, was recognized.	An iterative approach, to facilitate mutual learning in local communities, resulted in an increasingly broad scope and comprehensive objectives. High level of local commitment and collaboration indicated a strong alignment of target knowledge between initiative and all local actors and stakeholders.	Transformation knowledge integrated via collaboration with, and capacity building in, local hospitals. Education strategies for communities and schools aimed to foster transformation knowledge among general public. Production of manuals should allow replication of approach.
Strategic plan for implementing One Health, Kenya, from 2011^45^	Establishment of interministerial committees and task forces in charge of programme development, e.g. a national influenza task force, a zoonosis technical working group, One Health zoonotic disease units at central and peripheral levels and a One Health task force covering central and eastern Africa. Establishment of One Health offices within disease units and a national One Health secretariat.	Joint situation analyses of zoonotic diseases and the adoption of a One Health approach in routine and/or emergency activities fostered a shared understanding of systems knowledge.	Development of a One Health strategy/action plan strengthened common vision and direction at operational/institutional level. Inadequate funding for coordinated activities and lack of political will indicated insufficient alignment of objectives between initiative and high-level decision-makers.	A lack of institutional arrangements for coordination and collaboration between the line agencies and operational departments indicated that networks for collective action needed to be strengthened.
**Other initiatives**^a^				
Review of complex intersectoral services for child protection, the United Kingdom, 2010–2011^46^	Analysis of entire child-protection system to review and improve service provision at national level. Collaborative integration of evidence with stakeholders across entire chain of interests and responsibilities: affected individuals, charities, family proceedings courts, local institutions, national department of education and professionals.	Authorities and stakeholders jointly defined 60 relevant variables, and provided evidence on their relations, interactions and feedback loops. There was integration of systems knowledge through personal interactions, facilitated by joint building and analysis of system dynamics models. Group understanding developed in joint model analysis and validation.	Target knowledge was integrated via analysis of actor targets, as determinants of system behaviour.	Integration of transformation knowledge supported by joint definition and analysis of scenarios for transforming the activities and structures of the child-protection sector. Trust, networks and collaborative capacities for implementation were strengthened across hierarchies and sectors.
One Health surveillance and control, Canada, 2010–2012^47^	Analysis of integrated Lyme disease surveillance and control strategies to support decision-making and programme direction of public health authorities in Quebec. Collaboration with five national and regional authorities in agriculture, environment and public health. Actor perspectives on 11 strategic option’s effects on 16 target criteria were analysed under emerging and epidemic outbreak scenarios.	Focus groups, expert interviews and literature review facilitated integration of systems knowledge by joint problem definition and performance assessment of strategic options.	Target knowledge was integrated by defining targets in dialogue, discussion and reflection, by the elicitation and systematic analysis of stakeholder institution’s perspectives on target weights for animal, environmental and public, health, economic, operational, social and strategic impacts and surveillance, and by joint reflection on, and validation of the resulting multicriteria assessments.	Supported integrated transformation knowledge through joint elaboration of strategic options and target criteria, indicators and scales that were relevant to pertinent authorities. Participation in research, analysis and data analysis built collaborative capacities, networks for implementation and trust.
Intercultural collaboration for integrated health, Guatemala, 2012–2015^48^	Analysis of impacts, of a facilitated transdisciplinary approach, on trust, networks and mutual learning among biomedical doctors and traditional Maya healers. All in a country where structural violence hampers the development of integrative health systems. Collaborative referral designed to integrate different health systems in patients’ health-seeking pathways.	Integration of systems knowledge facilitated among practitioners via joint design and validation of empirical research on barriers to integrative health services. Group understanding was developed through workshop techniques and the changes in perspective that occurred during joint fieldwork.	Integration of target knowledge was supported through increased understanding of the perspectives of other participating actors and via negotiation of the characteristics and objectives of the transdisciplinary approach.	Integration of transformation knowledge was supported by strengthening collaborative capacities, by an improved understanding of viewpoints of other actors which in many other projects remain hidden because of the segregation of institutions and sectors, and by the joint development, implementation, and assessment of pilot models for institutional and operational transformation.

^a^ Initiatives that applied specific knowledge integration approaches in fields of relevance to One Health.

### Integration of target knowledge

The integration of target knowledge has been fostered by including stakeholder perspectives in agenda setting and decision-making, through either explicit co-leadership and negotiation^43^^,^^45^^,^^48^ or changes of perspective in collaborative work assignments.^44^^,^^46^^,^^48^ In Quebec, Canada, a rigorous multicriteria analysis, of Lyme disease surveillance and control strategies was used to support the public health authorities’ decision-making and programme direction.^47^ In the latter investigation, a participative approach that involved health professionals and other stakeholders from governmental and nongovernmental organizations was used to compare several surveillance strategies in terms of their likely animal, environmental and public health and socioeconomic impacts. The stakeholder group provided input during the definition of management strategies, the assessment of objectives and their relative importance and the scoring of the strategies in terms of their likely attainment of the objectives. Since stakeholders represented their institutional perspectives, the process presumably assured the balanced representation of each of the relevant institutional viewpoints. The analyses allowed preference rankings of several possible intervention strategies for the management of Lyme disease, facilitated a better understanding of the conflicts between the key objectives and the relevance of such conflicts to each stakeholder group, and apparently improved each stakeholder group’s appreciation of the preferences and priorities of the other stakeholder groups. In short, the analyses contributed to resolving trade-offs and setting a common vision and direction. While multicriteria analyses have mostly been focused on the early stages of policy development, e.g. agenda setting and policy formulation, they have important evaluative elements and can build consensus, to strengthen collective action, during policy implementation (Table 1).

### Integration of systems knowledge

The integration of systems knowledge has been used in the joint definition of broad conceptual bases for the collection and assessment of evidence^43^^,^^45^^,^^47^^,^^48^ and in facilitating group understanding of the evidence collected via collaborative data analysis and validation (Table 1).^43^^,^^45^^,^^47^ In the United Kingdom of Great Britain and Northern Ireland, a comprehensive intersectoral review of the activities, culture, effectiveness, policies and social relations within the child-protection sector demonstrated how One Health governance could be supported by structured and rigorous systems thinking.^46^ This review engaged a reference group of relevant stakeholders, e.g. representatives from charities, the civil service and other government departments, an adoptive mother and young people who had been through the child-protection system themselves, and drew on evidence from databases, written sources and individual stakeholders’ perspectives. The collaborative development of causal loop diagrams, with 60 variables, facilitated both a better understanding of the systemic outcomes of interdependent decision-making processes and a comparative assessment of potential policy interventions. The recommendations drawn from this review’s results were largely accepted by the commissioning government authority and triggered substantial policy changes.^46^ Systems thinking can therefore transform complex mixtures of individual observations into coherent narratives that state how situations emerge and how they may unfold in the future. While systems thinking has mostly focused on the evaluation stage of policy development, it usually includes target knowledge, as a determinant of behaviour, and its participative nature can also build trust and foster mutual learning between stakeholders and scientists.

### Integration of transformation knowledge

Most One Health and related initiatives rely on a multi-institutional network of actors. This network often contributes to the integration of transformation knowledge in two ways: via the institutional support provided by relevant decision-makers^43^^,^^46^^,^^47^ and via the collaboration of individuals who have a broad range of implementation-related skills and expertise in many specialist fields.^43^^,^^44^^,^^46^^,^^47^ The potential usefulness of transdisciplinary approaches for coordinating and managing such interdisciplinary, intersectoral and intercultural collaboration, even in challenging societal contexts, was illustrated by a collaboration in Guatemala.^48^ The main aim of this collaboration was to bridge the gaps between the knowledge systems of biomedical doctors and those of traditional Maya healers and, in so doing, promote collaboration and mutual learning between the two groups. After facilitating joint patient diagnosis and subsequent treatment reconstruction, the collaboration was deemed useful and relevant by both groups of subjects and appears to have reduced the long-standing prejudices held by each group towards the other. Scientific institutions that, in terms of these prejudices, were perceived as neutral acted as intermediaries and helped ensure the credibility of the results. The process provided multiple opportunities for the building of mutual trust, via dialogue and experiential exchange and also triggered reflection, by pointing out the shortcomings of the current health systems, and appears to have educated all of the participants. In short, it developed and/or strengthened the networks for collective action. While the Guatemalan study focused on the implementation stage of policy development, the transdisciplinary approaches also had effects on agenda setting, by influencing the actors’ target knowledge and on evaluation, by enabling process assessments that were more inclusive of the divergent knowledge systems (Table 1). Furthermore, the new networks and increased levels of trust helped achieve consensus and collective action at every stage of policy development.

## Discussion

**Fig. 1:**
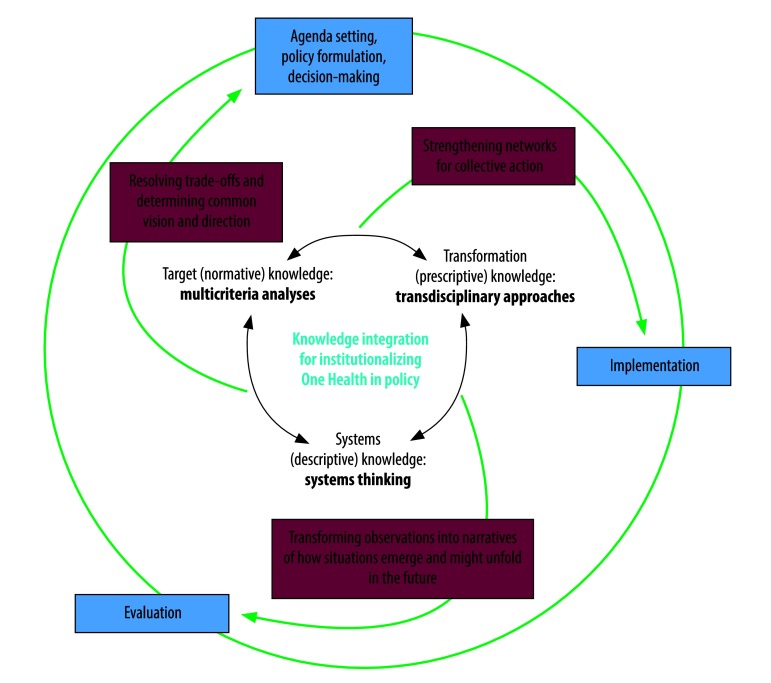
Potential uses of knowledge integration within One Health policy cycles

Notes: The outer and inner circles indicate the stages within each round of policy development^21^ and the approaches for integrating target, transformation and systems knowledge,^28^ respectively. The text on the arrows summarizes the main benefits of knowledge integration for each stage of policy development.

We believe that knowledge integration is both an integral element of successful One Health governance systems and a prerequisite for the effective implementation of the One Health approach. The combined use of multicriteria analyses, systems thinking and transdisciplinary approaches (Fig. 1) could contribute to more systematic and successful collaborations within and across existing institutions and form a procedural backbone for converting the aspirations of the One Health concept into institutional processes. In general, the aim of multicriteria analyses, systems thinking and transdisciplinary approaches is to create, maintain and inform collective action by broad coalitions of societal partners. If successfully implemented over extended time spans, they could contribute to the building of trust, networks and institutions that are not primarily dependent on any existing hierarchical structures of government.

Although multicriteria analyses, systems thinking and transdisciplinary approaches mainly focus on different, crucial aspects of One Health governance, they are complementary and overlapping rather than mutually exclusive. They provide methods to resolve trade-offs and set a common vision and a common direction across disciplines, institutions and sectors. They serve as a toolbox for systemic monitoring and feedback to transform observations into narratives detailing how situations emerge and might unfold in the future. Finally, they contribute to the development and/or strengthening of networks for collective action towards a common vision. Potentially, therefore, as a decisive element in policy development, knowledge integration could help resolve the main shortcomings of the current global framework for health governance, by managing complexity and shaping interactions between actors and institutions towards joint learning.^17^^,^^18^ Knowledge integration could also be used to complement educational and institutional measures for improving the implementation of the One Health approach.^14^ We therefore propose that policy cycles relevant to One Health should aim at knowledge integration and make the best possible use of multicriteria analyses, systems thinking and transdisciplinary approaches. Whenever they are used as elements of the implementation of the One Health approach, the processes involved in knowledge integration should be reported explicitly in the associated scientific articles. Ideally, such reporting should be based on standardized criteria and systematic evaluation frameworks, like the one proposed by the Network for Evaluation of One Health.^5^^,^^20^ To develop and improve best practices in One Health, the practitioners and scientists in active One Health networks should be educated on knowledge integration and encouraged to discuss their ideas with those of more established governance actors, ideally in programmes supported by permanent professional associations or organizations. Finally, attention should be directed towards developing and implementing efficient technical mechanisms to facilitate stakeholder involvement and brokering at all levels of health governance, from local to global level.
